# Transcriptome Analysis of Long-lived *Drosophila melanogaster E*(*z*) Mutants Sheds Light on the Molecular Mechanisms of Longevity

**DOI:** 10.1038/s41598-019-45714-x

**Published:** 2019-06-24

**Authors:** Alexey A. Moskalev, Mikhail V. Shaposhnikov, Nadezhda V. Zemskaya, Liubov А. Koval, Eugenia V. Schegoleva, Zulfiya G. Guvatova, George S. Krasnov, Ilya A. Solovev, Maksim A. Sheptyakov, Alex Zhavoronkov, Anna V. Kudryavtseva

**Affiliations:** 1 Engelhardt Institute of Molecular Biology, Russian Academy of Sciences, Moscow, Russia; 2 Institute of Biology of Komi Science Center of Ural Branch of RAS, Syktyvkar, Russia; 3 Moscow Institute of Physics and Technology, Dolgoprudny, Russia; 4 Insilico Medicine, Rockville, USA

**Keywords:** Ageing, Drosophila

## Abstract

The *E*(*z*) histone methyltransferase heterozygous mutation in *Drosophila* is known to increase lifespan and stress resistance. However, the longevity mechanisms of *E*(*z*) mutants have not been revealed. Using genome-wide transcriptome analysis, we demonstrated that lifespan extension, increase of resistance to hyperthermia, oxidative stress and endoplasmic reticulum stress, and fecundity enhancement in *E*(*z*) heterozygous mutants are accompanied by changes in the expression level of 239 genes (p < 0.05). Our results demonstrated sex-specific effects of *E*(*z*) mutation on gene expression, which, however, did not lead to differences in lifespan extension in both sexes. We observed that a mutation in an *E*(*z*) gene leads to perturbations in gene expression, most of which participates in metabolism, such as Carbohydrate metabolism, Lipid metabolism, Drug metabolism, Nucleotide metabolism. Age-dependent changes in the expression of genes involved in pathways related to immune response, cell cycle, and ribosome biogenesis were found.

## Introduction

Gene expression fluctuates in different tissues and the chromatin landscape changes during aging^1^. Site-specific repression of the genome is known to be observed in aged individuals, as well as multifocal (or global) derepression, the latter process dominates^2,3^. Gene expression is usually influenced by the chromatin state, but the transcriptional profile also may influence epigenome, especially when sequences regulating chromatin-remodeling complexes are affected^4,5^. The lowering of age-related gene expression profiles is known to be associated with heterochromatin formation and histone methylation. This statement is true for histone H3 lysine 9 (H3K9) and histone H3 lysine 27 (H3K27) loci in *Drosophila* and is connected to the pool of histone methyltransferases^3^. The H3K9 age-related loss results in the Heterochromatin Protein 1 (HP1) concentration lowering, this phenomenon is strongly associated with heterochromatin decompactization and genomic instability^6^. In addition, the epigenome-wide loss of H3K9 methylation has been observed in cell culture modeling Hutchinson-Gilford progeria syndrome and also in aged *Drosophila melanogaster*, as an exception, several chromatin islands are hypermethylated in both cases^3,6,7^.

Histone H3 lysine 9 methylation is carried out by dSETDB1, Su(var)3–9, G9a, and E(z)^8,9^. The H3K27 methylation levels are associated mainly with E(z) and ESC subunits of polycomb repressive complex 2 (PRC2). Wild type *E*(*z*) (*Enhancer of zeste*) is a gene coding protein that belongs to the class V-like SAM-binding methyltransferase superfamily, histone-lysine methyltransferase family, and EZ subfamily. E(z) is also known to have transcription factor activity^5,10^.

The loss-of-function mutations in PRC2 components extend the lifespan^11^ and take part in transgenerational inheritance mechanism detected for this effect, as well as the antagonistic enzyme UTX-1 demethylating H3K27me3^12^. PRC2, and especially its subunit E(z), are known as suppressors of stress-response genes (e.g. *Odc1*). This complex is working in an ensemble with trithorax (TRX), which has the opposite function^11^. In the present work, we aim to unravel specific mechanisms which cause life extension, fecundity augmentation, and improve stress-response in *E*(*z*) mutants through the use of full transcriptome analysis. We observed a perturbation effect of *E*(*z*) mutation on the expression level of genes involved in Carbohydrate metabolism, Lipid metabolism, Drug metabolism, and Nucleotide metabolism. We also found sex-specific effects of *E*(*z*) mutation on the age-dependent changes in the expression of genes involved in pathways related to the immune response, cell cycle, and ribosome biogenesis. In addition, we demonstrated that the *E*(*z*) mutation affect a plethora of poorly described *Drosophila* genes. The functions of these genes in lifespan determination are not understood and may not be associated with aging and longevity.

## Results

### Lifespan

**Figure 1:**
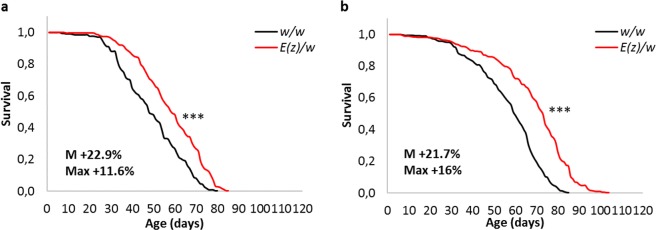
Influence of heterozygous mutation in *E*(*z*) on male (**a**) and female (**b**) lifespan (the results of 3 replicates are pooled). M, Max - increase of median and maximum lifespan, respectively. ***p < 0.001, Kolmogorov-Smirnov Test.

In earlier work, Siebold *et al*. (2010) showed that *E*(*z*) heterozygous mutants were characterized by reduced H3K27me3 levels^11^. Although homozygous null mutations in *E*(*z*) cause earlier lethality in embryos^13^, in *E*(*z*) heterozygous mutant phenotype, median lifespan is prolonged by 71–76% compared to short-living *Oregon-R* control line, and by 33% compared to long-living *Canton-S*^11^. Our research reproduced the *E*(*z*) heterozygous mutation’s effect on lifespan extension (Fig. 1 and Table 1). We observed an increase in median lifespan by 22.9% in male *E*(*z*)/*w* mutants and by 21.7% in females compared to long-living *w*/*w* control strain. The maximum lifespan of *E*(*z*)/*w* was also elongated by 11.6% in males and by 16% in females.

**Table 1:** Influence of heterozygous mutation in *E*(*z*) on lifespan parameters.

Variant	Sex	Replicates	M (days)	dM (%)	Log-Rank Test (p)	90% (days)	d90% (%)	Wang-Allison Test (p)	MRDT (days)	dMRDT (%)	n
*w*/*w*	♂	1	45			67			10		96
*E*(*z*)/*w*	♂	1	59	31.1	p < 0.0001	78	16.4	p < 0.0001	9.9	−1	109
*w*/*w*	♂	2	48			69			10.7		111
*E*(*z*)/*w*	♂	2	58.5	21.9	p < 0.005	76	10.1	p < 0.0001	9.4	−12.1	84
*w*/*w*	♂	3	53			71			9.4		136
*E*(*z*)/*w*	♂	3	59	11.3	p < 0.001	74	4.2	p < 0.001	8	−14.9	85
*w*/*w*	♂	Pooled	48			69			10.2		343
*E*(*z*)/*w*	♂	Pooled	59	22.9	p < 0.0001	77	11.6	p < 0.0001	9.1	−10.8	278
*w*/*w*	♀	1	56			74			9.8		159
*E*(*z*)/*w*	♀	1	73	30.4	p < 0.0001	91	23	p < 0.0001	11.7	19.4	131
*w*/*w*	♀	2	56			72			9.6		151
*E*(*z*)/*w*	♀	2	74	32.1	p < 0.0001	88	22.2	p < 0.0001	9.8	2.1	142
*w*/*w*	♀	3	64			77			7.7		217
*E*(*z*)/*w*	♀	3	71	10.9	p < 0.0001	86	11.7	p < 0.0001	7.5	−2.6	127
*w*/*w*	♀	Pooled	60			75			9		527
*E*(*z*)/*w*	♀	Pooled	73	21.7	p < 0.0001	87	16	p < 0.0001	9.9	10	400

♂ - male; ♀ - female; M - median lifespan; 90% - age of 90% mortality (maximum lifespan); MRDT - mortality rate doubling time; dM, d90%, dMRDT - differences between median lifespan, age of 90% mortality, and MRDT of the control and experimental flies, respectively; n - number of flies. To compare the statistical differences in median and maximum lifespan between the control and experimental groups, the Log-Rank and Wang-Allison tests were used, respectively. The results of 3 replicates are pooled and presented as survival curves in Fig. 1.

### Stress resistance

In *E*(*z*) mutants, an increase in resistance to oxidative stress and starvation was observed by Siebold *et al*.^11^. The loss of H3K27 methylation in *E*(*z*) leads to precise derepression of PRC2 targets. Gene *Odc1*, coding ornithine decarboxylase, which is essential for polyamine synthesis, is one of them. Polyamines are involved in oxidative stress response, and these compounds extend lifespan in nematodes, flies, and mice^11^.

**Figure 2:**
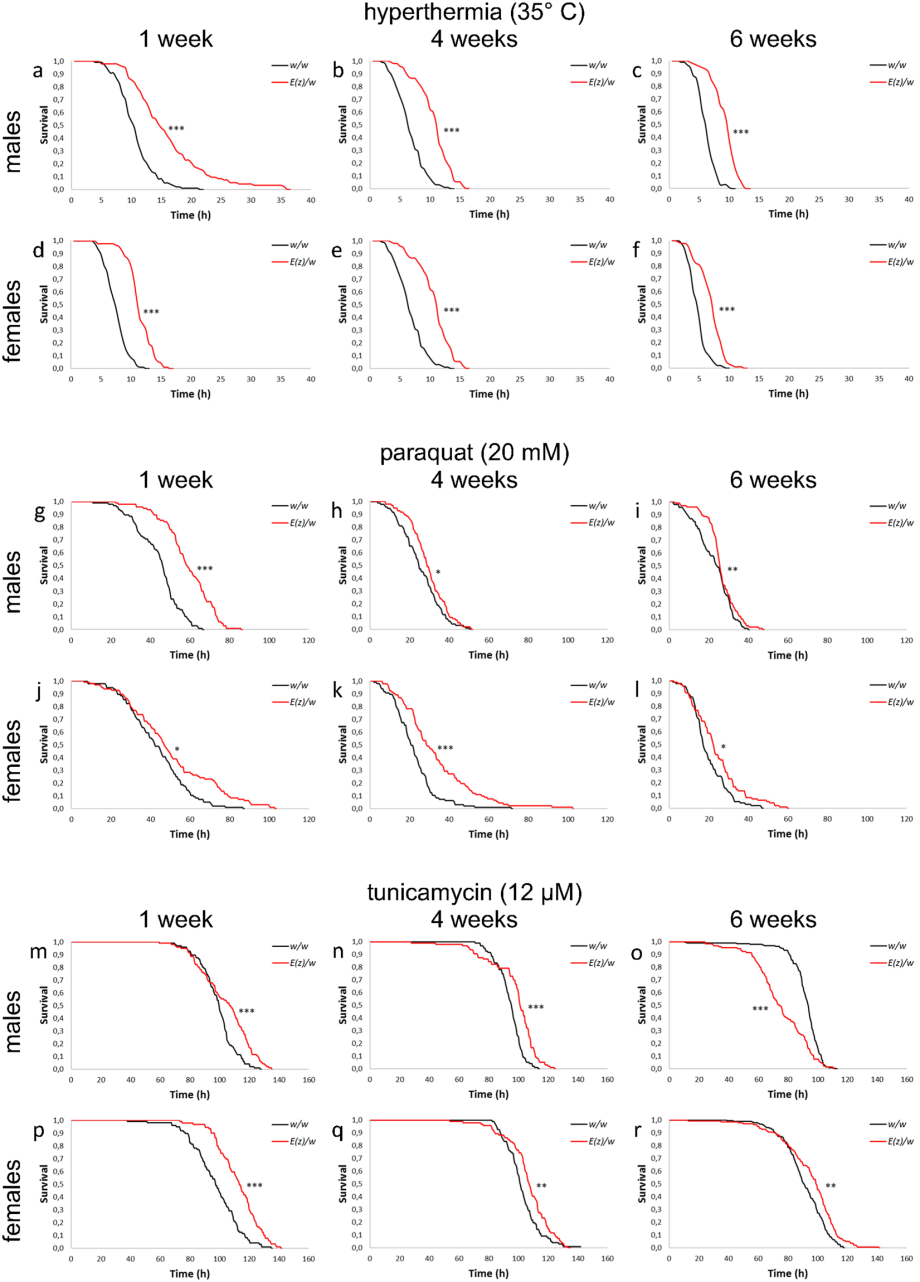
Survival of *E*(*z*) mutants in different ages under stress conditions. *p < 0.05, **p < 0.01, ***p < 0.001, Kolmogorov-Smirnov Test.

As far as the correlation of resistance to stress with lifespan, we evaluated the effect of the *E*(*z*) mutation on survival in conditions of hyperthermia, oxidative stress, and endoplasmic reticulum (ER) stress (Fig. 2, Supplementary Fig. 1 and Supplementary Table 1). We observed an increase (p < 0.001) of survival time percentiles under heat stress (35 °С) in males (by 42–100%) and females (by 28–50%) carrying the *E*(*z*) gene mutation relative to the control at the ages of 1, 4, and 6 weeks (Supplementary Fig. 1a,b, Supplementary Table 1). The survival curves also show differences (p < 0.001) between *E*(*z*) mutants and the control line (Fig. 2a–f).

In individuals with an *E*(*z*) mutation, an increase in resistance to paraquat was found (Fig. 2g–l, Supplementary Fig. 1c,d, Supplementary Table 1). After the first week of life, an increase in survival time percentiles was observed in males (by 25–48%), compared to the control (p < 0.001). At the age of 4 and 6 weeks, only the 25^th^ percentiles of survival time were higher in *E*(*z*)/*w* males (by 30–40%) as compared to the control (p < 0.01). Mutant *E*(*z*) females at the age of 1 week were characterized by an increase of 90^th^ percentiles by 22%, relative to the control group (p < 0.05). At the age of 4 weeks, a significant increase was observed of all survival time percentiles by 30–40%. At the age of 6 weeks an increase of the 50^th^ and 75^th^ percentiles by 22% and 10% (p < 0.05), respectively, was found in *E*(*z*)/*w* females. Thus, the oxidative stress-resistance improvement takes place in *E*(*z*) mutants at all ages.

Genotype *E*(*z*)/*w* determines enhancement in resistance to ER stress induced by tunicamycin (Fig. 2m–r, Supplementary Fig. 1e,f, Supplementary Table 1). Only the survival in males on the 6^th^ week was 6–28% lower, compared to the control group (reproduced in six replicates). The growth in survival parameters was registered after one week of a male imago’s life; the positive effect was revealed in the 75^th^ and 90^th^ percentiles (about 10% increase), and after four weeks, the same effect was found in 50^th^, 75^th^ and in 90^th^ percentiles (p < 0.05). *E*(*z*) mutant females were more resistant to ER stress, especially on the first week (10–14% increase, p < 0.001); on the fourth and sixth weeks, they were 6–8% more resistant to ER stress compared to the control cohort (p < 0.01).

Therefore, the *E*(*z*) mutation improves *D*. *melanogaster* survival in acute stress conditions of a different modality (hyperthermia, oxidative stress, ER stress), showing a somewhat positive genetic alteration for fruit flies.

### Fecundity

**Figure 3:**
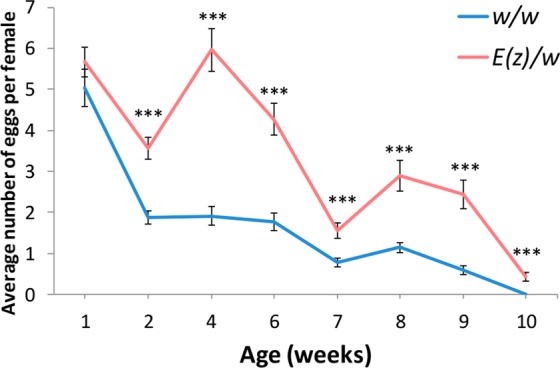
Age-dependent dynamics of fecundity. The error bars show standard errors. ***p < 0.001, t-Student test.

Female flies carrying *E*(*z*) mutation demonstrated higher fecundity compared to the control cohort of *w*/*w*. In the first week, statistically significant differences were not observed, thus it was an exception. However, in *E*(*z*) flies, the number of laid eggs was larger, which saves the general tendency presented in Fig. 3.

In *Drosophila*, lifespan extension induced by overexpression of pro-longevity genes, or mutations in anti-longevity genes, is frequently associated with decreased reproduction^14^. However, overexpression of *FOXO* in head and in fat body, overexpression of *D-GADD45* in the nervous system, and mutation in *Indy* have been shown to increase lifespan without a decrease in fecundity^14–17^.

We demonstrated that *E*(*z*) mutation disturbs the longevity-reproduction trade-off. Differential gene expression analysis revealed a decreased expression level of some genes that code for the structural components of the vitelline membrane (*Vm32E*, *Vm26Ac*, *Vm26Aa*, *Vm34Ca*, *Vml*, *Vm26Ab*) in *E*(*z*) mutants despite the high fecundity. But only *Vm32E* and *Vm26Ac* passed the p-value threshold (Supplementary Table 2).

### Transcriptome analysis in *E*(*z*) mutants

It was previously shown that the increase in the lifespan of *E*(*z*) mutants was observed only in the heterozygotic imagoes, characterized by the incomplete inhibition of *E*(*z*) expression^11^. To explore the mechanisms of longevity in mutants, an analysis of whole transcriptome changes was performed against the background of the normal activity of this gene (*w*/*w*) in comparison with the strain that has partial *E*(*z*) suppression (*E*(*z*)/*w*). Flies with an aligned genetic background were used in the experiment. To align the genetic background, the line *w*^*1118*^ was used.

Genome-wide transcriptome analysis identified 859 genes with expression level changes that are associated (СPM < 0.1, p < 0.05) with a mutation in a *E*(*z*) gene. Of those genes, 239 showed changes in the expression of more than 2 times (77 genes passed FDR < 0.05 threshold) (Supplementary Table 4). Among the most differentially expressed genes that showed an increase in expression (LogFC > 2, FDR < 0.05), the following can be identified: *Unc-115b*, *p24-2*, *tobi*, *Ir76a*, *CG13313*, *CG3397*, *CG4098*, *CG34031*, *CG12224*, and *CG13460*. The genes showing a decrease in expression include (LogFC < −2, FDR < 0.05) *CG32379*, *CR44138*, and *CG42857* (Supplementary Table 5).

The most differentially expressed gene found was *tobi* (*target of brain insulin*). Tobi is a protein that has hydrolase activity and because it hydrolyzes O-glycosyl compounds, it improves carbohydrate metabolic processes^18^. A great variety of O-glycosyl compounds are proteins and lipids that have been glycated as a result of exposure to sugar solution. These compounds are also called advanced glycation end products (AGEs). AGEs are known for their pro-aging effects and for their exacerbation of the course of degenerative diseases, e.g. Alzheimer’s disease, diabetes, atherosclerosis, etc^19^. However, transgene overexpression of *tobi* has been shown to reduce *Drosophila* growth and viability^18^ and the role of this gene in longevity is not unique.

A immense increase was observed in the *Unc-115b* gene, coding a Zinc-finger, LIM type domain protein expressed mainly in imago’s brain tracts, especially in the glial sheath and in the system of fibers. *Unc-115b* is expressed in the superficial layer of the fly’s brain called the perikaryal rind. The expression is localized in nucleoplasm and associated with cytoskeleton organization^20^.

Gene *p24-2* is one of the highest peaks in the obtained transcriptional profile. The protein p24-2 has a membrane single-pass type-I transporter activity and is associated with Golgi-vesicle transport. It is also known that *p24-2* regulates post-mating oviposition, therefore, it is a candidate-gene to determine the effects found in the fecundity measurements of *E*(*z*) mutants^21,22^.

The next sequence that is upregulated in *E*(*z*) mutants is *CG13313*. Little data may be found to describe this protein-coding gene, and the spectre of its functions is still unexplored. The highest expression *CG13313* is associated mainly with adult malpighian tubules and in *Drosophila* midgut. CG13313 protein, according to InterPro analysis, carries CUB domain, which is included in spermadhesin, CUB-domain superfamily. In human, it is called Complement C1S component. The role of spermadhesin varies greatly in other mammals. Interestingly, the concentration of *CG13313* mRNA grows gradually in male flies with age^23^.

A gene *CG3397* codes an aldo/keto reductase and NADP-dependent oxidoreductase that may be involved in the neutralization of reactive oxygen species and a neuronal defense mechanism against oxidative stress^24^. This gene may be involved in stress resistance of *E*(*z*) mutants.

The ionotropic receptor 76a encoded by *Ir76a* gene is described as an olfactory receptor molecule that detects chemical stimuli and is responsible for the sensory perception of smell^25^. In *Drosophila*, the mutation of odorant receptor Or83b has been shown to cause severe olfactory defects, alter adult metabolism, enhance stress resistance, and extend lifespan^26^, but the function of *Ir76a* in longevity determination is unknown.

Among downregulated genes, the *CG32379* codes product with metallocarboxypeptidase activity. The functions of *CR44138* and *CG42857* are unknown. None of these downregulated genes have been described in connection with aging and longevity.

**Figure 4:**
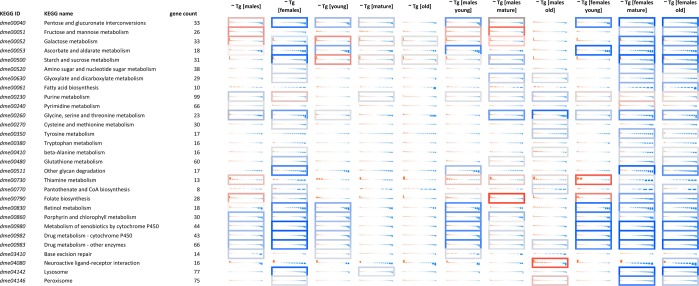
Expression level changes induced by *E*(*z*) mutations in various subgroups (young/mature/old, males/females). Each cell represent the sorted binary logarithms of expression value fold changes (LogFC) in *E*(*z*) mutants versus control species for genes participating a specific KEGG pathway. LogFC (vertical axis) is ranged from −2 to +2, i.e. from 4-fold decrease (blue) to 4-fold increase (red). Cell borders demonstrate the statistical significance of gene set enrichment analysis (Fisher test *p*-value): blue (enriched with downregulated genes) and red (enriched with overexpressed ones). The present data include only genes with average read counts per million (CPM) greater than 8.

Reactome pathway analysis demonstrated that the expression of biological oxidation pathways’ elements decreased in mutant flies of all age groups of both sexes (Fig. 4). In females of all age groups, a decrease was shown in the expression levels of genes involved in arachidonic acid metabolism. Genes involved in the regulation of N-glycosylation and in the activity of neurons had increased expression with the largest contribution in the “mature” and “old” *Drosophila* cohorts (both in males and females). In general, the analysis of Kyoto Encyclopedia of Genes and Genomes (KEGG) pathways demonstrated the inhibition of genes involved in ascorbate and aldarate, retinol, porphyrins, xenobiotics, serine, and glycine metabolism.

Among the age-related transcriptomic changes, the downregulation of ribosome components and ribosome biogenesis could constitute a hallmark of ageing^27^. Among age-dependent DE genes (both among control and experimental flies) we found that most of the genes (according to KEGG pathway and GO terms) are reduced in females (Supplementary Fig. 7). Interestingly, comparing old *E*(*z*)/*w* females with the control flies, we found that the expression for most of the genes related to ribosome biogenesis was higher in mutant flies than in control ones (Supplementary Fig. 12). On the contrary, in males, the expression level of genes in old mutant flies is lower than in control ones (Supplementary Fig. 10), while with age, an increased expression of the genes of this pathway were detected. (Supplementary Fig. 4).

According to Frenk *et al*. (2018) reduction in growth factor signaling is one of the gene expression hallmarks of ageing^27^. Our data demonstrates that about 80% of DE genes in functional categories associated with cell cycle and DNA replication were age-related increased in females and about the 60% of the genes were increased in males (Supplementary Figs 5 and 8). A number of studies revealed the expression of genes associated with the cell cycle and DNA replication was reduced in ageing mice^28,29^.

It is known that the expression of immune response genes is constantly increased with age in *Drosophila*^30–32^. We found changes in the expression of genes associated with this GO term (Supplementary Figs 3 and 6). Comparing long-lived *E*(*z*) mutants with the control flies in the “old” group, we found that the expression of more than 70% of genes related to GO terms for immune response, such as innate immune response, inflammatory response, Toll and Ind signaling is reduced in *E*(*z*) mutants (Supplementary Figs 9 and 11). At the same time, we found the downregulation of proteases and cytochrome p450, for which previously detected higher expression in long-lived *Drosophila* and downregulation during the response to inflammation or infection^33^. On the other hand, Cytochromes P450 enzymes alters its level of expression in tissue-, and time-dependent manner^34^.

We found a significant decline in the expression of Turandot family genes (*TotC*, *TotM*, *TotX*, *TotA*) in mutant flies of both sexes at the age of 4 and 6 weeks, while we observed an increase in the expression of these genes in flies at the age of 1 week (Supplementary Table 6). It is known that the genes of the Turandot family are regulated by JAK-STAT and MAPK pathways and are induced in response to various types of stress, including starvation, bacterial and fungal infections, irradiation, heat shock, oxidative stress, and the aging process^35–37^. It is possible to assume that the Turandot family suppression in our case is associated with changes in the stress response formation in mutant *E*(*z*) background, causing stress load diminishing. DNase SID takes part in apoptotic DNA fragmentation and is downregulated in *E*(*z*) mutants (Supplementary Table 7), *Sid* is known to be induced by bacterial infection and oxidative stress^38^. The *Sid* suppression may determine the decrease in DNA fragmentation that improves genome integrity showing a pro-longevity factor.

Besides, we also found that genes of the family of antimicrobial peptides (AMP) showed a sex-specific difference in expression. Interestingly, in a group of males, the expression of the attacine (*AttA*, *AttB*, *AttC*) and diptericin (*DptA*, *DptB*) genes decreased, and in the female group, this tendency took place only in “young” flies. For groups of females 4 and 6 weeks, an increase in the AMP expression was found. The cecropin genes (*CecA2*, *CecA1*, *CecC*) in females show an increase in expression profiles in all age groups, while in the male group we found a decrease in the expression level of these genes. It is also worth noting that the changes are much more pronounced in groups of “old” individuals. An earlier analysis of the aging-associated changes in the transcriptome revealed a significant increase in the level of expression of AMP genes in aging flies^39,40^. It was also noted that the level of AMP expression in young flies correlates negatively with life expectancy^40^. In addition to antimicrobial activity, some AMP genes are involved in proliferation, wound healing, chemotaxis, and anti-apoptosis. Zhao *et al*. (2010) also showed that Dpt and Att play a role in tolerance to hyperoxia^41^.

Comparing the genes which were strongly over-represented among H3K27me3-marked genes (according to literature data) with a list of differentially expressed genes in the *E*(*z*) mutant we found only 3 genes that showed statistically significant changes in expression level in the *E*(*z*) mutant^42–45^. These are *Drosophila* ortholog of *developing brain homeobox* (*Dbx*), *Ultrabithorax* (*Ubx*), and *CG34031* genes, which belong to the homeobox (Hox) gene family. All other genes failed to pass the p-value threshold. We observed a slight increase in the expression of *Ubx* gene, while the *Dbx* gene showed a 2.8-fold decrease in the expression level. Homeotic protein ultrabithorax targets hundreds of different genes including regulatory genes, such as transcription factors, signaling components, and terminal differentiation genes^46–48^ and its repression is regulated through trimethylation at H3K27 and H3K9^49^. The Dbx is a homeodomain transcription factor and involved in the development of specific subsets of interneurons in flies and vertebrates^50–52^. In vertebrates, Dbx participates in the regulation of proper left-right alternation of motoneuron firing^53^. Lacin *et al*. (2009) showed that *Dbx* mutant flies exhibit defects in locomotor functions, such as dysregulation of flight and walking movements^52^. Despite the downregulation of the *Dbx* gene, we did not observe a decrease in the locomotor activity of the *E*(*z*) mutants compared with the control line *w*/*w*. Contrary, in the age of four weeks, the locomotor activity of *E*(*z*)/*w* males was higher than in *w*/*w* (Supplementary Fig. 2). Homeobox domain motif-carrying gene *CG34031* encodes a transcriptional regulator having sequence-specific DNA binding activity^54^. Previously, it has only been described by Xu *et al*. (2014) as a Tip60-inducible gene (Tip60 is a histone acetyltransferase causing chromatin relaxation)^55^.

## Discussion

In the present research we studied the effects of *E*(*z*) histone methyltransferase heterozygous mutation on lifespan, stress-resistance, fecundity, and genome-wide transcriptional profile dynamics in *Drosophila* imagoes. We observed 22–23% lifespan extension in both sexes, and *E*(*z*) mutants were significantly more resistant to hyperthermia, oxidative stress and endoplasmic reticulum stress, and demonstrated enhanced fecundity.

The genome-wide transcriptome analysis identified 239 genes (p < 0.05), which expression level was altered more than 2 times by *E*(*z*) mutation. Several of the most differentially expressed genes had never been described before as pro-longevity genes. Most likely, these DE genes may be associated with *E*(*z*) mutation, but not related to aging and longevity. Among them, the following upregulated genes were identified: *Unc-115b*, *p24-2*, *tobi*, *Ir76a*, *CG13313*, *CG3397*, *CG4098*, *CG34031*, *CG12224*, and *CG13460*. A decreased expression was found for: *CG32379*, *CR44138*, and *CG42857*. The exception is a differential expression of antimicrobial peptides (AMP) and the Turandot family mentioned above. These humoral innate immunity factors have been previously discussed in the context of aging and stress-resistance^36,39,40^.

A mutation in the *E*(*z*) gene surprisingly neither activated nor repressed canonical pro-longevity or anti-longevity genes like mTor or insulin/IGF-signaling elements and either determinants of DNA-repair, *Sod*, Sirtuins, etc. We also did not find strong changes in the expression of the Hox family of genes, for which gene repression by polycomb group proteins was previously shown^56^.

As can be seen from Fig. 4, a mutation in an *E*(*z*) gene leads to perturbations in pathways, which are mostly related to metabolism. Here our studies agree with Brookes *et al.* (2012) which also showed that the genes marked by PRC have roles in metabolism^57^.

We observed that the *E*(*z*) mutation leads to modulation of many genes related to the immune response, ribosome biogenesis, and cell cycle (Supplementary Figs 3–12). Although age-dependent changes in the expression of these genes are similar to changes in control flies, it is most likely that mutations in *E*(*z*) lead to positive perturbations in the pathways for which age-associated gene expression changes are shown^27^. In addition, as indicated in heatmaps (Supplementary Figs 3–12) and Fig. 4, the gene expression is sex-specific, despite the fact that the increase in median lifespan for both sexes was broadly similar, which emphasizes the importance of separate preparation and analysis of females and males. Turning our attention to the KEGG pathway analysis, we noted a strong sex-specificity of pathway enrichment. For example, genes involved in control of fructose, mannose, galactose metabolism, as well as glycerolipid and fatty acid metabolism were mainly upregulated in male *E*(*z*) mutants, in addition to the neuroactive ligand-receptor interaction pathway. Genes controlling purine and pyrimidine metabolic pathways were activated in female mutant flies, as well as the RNA polymerase-associated group of genes. The trend of strong pathway downregulation was registered more frequently in female flies, meaning that the majority (not all) of pathway-associated genes decreased expression.

A holistic view of the present work implies a construction of a complicated transcriptional landscape that is formed by site-specific (H3K9 and H3K27) loss of histone methylation. On one hand, the global derepression is a deleterious process. On the other hand, the precise derepression of specific genes found and activity modulation of metabolic pathways show significant anti-aging effect and healthspan extension. The results obtained in the work dispose us to accept the hypothesis that site-specific global derepression does not cause genome instability, aberrant transcription, transcriptional drift, and total expression profile uplifting as was previously reviewed in Solovev *et al*.^3^. In fact, it can even extend lifespan, or improve stress resistance and fecundity.

## Methods

### *Drosophila* lines and crosses

*Drosophila melanogaster w*^1118^ (#3605, Bloomington *Drosophila* Stock Center, USA), *E*(*z*)^+^/*TM6C* (balancer line with wild-type *E*(*z*) allele) and *E*(*z*)^*731*^/*TM6C* (#24470, Bloomington *Drosophila* Stock Center, USA; possesses *E*(*z*) loss-of-function allele^58^) were used in interstrain crosses to obtain flies for control and experimental cohorts. In order to match the genetic background of *E*(*z*)^*731*^/*TM6C* and *E*(*z*)^+^/*TM6C* lines, they were backcrossed into *w*^*1118*^ eight times. All experiments (analysis of lifespan, stress resistance, fecundity, and RNA sequencing) were performed with the F_1_ progenies of *w*^*1118*^ females and backcrossed *E*(*z*)^+^/*TM6C* or *E*(*z*)^*731*^/*TM6C* males. The *E*(*z*)^+^/*w*^1118^ (hereafter *w*/*w*) offspring were used as a control and *E*(*z*)^*731*^/*w*^1118^ (hereafter *E*(*z*)/*w*) as an experimental flies.

### Lifespan analysis

Control and experimental flies were collected within 24 hours of eclosion, sorted by sex under carbon dioxide (CO_2_) anesthesia (Genesee Scientific, USA), and maintained in a climate chamber Binder KBF720-ICH (Binder, Germany) on a food medium containing 1000 mL water, 7 g agar, 8 g yeast, 30 g sugar, 30 g semolina, and 3 mL propionic acid at constant temperature (25 °C) and humidity (60%) in a 12 hours:12 hours light:dark schedule. The flies were housed in *Drosophila* narrow vials (Genesee Scientific, USA) at a density of 30 individuals per vial, with 3–5 vials per experimental variant. Experiments were performed in three replicates (a total of 270–340 males and 400–500 females were analyzed). Fresh medium vials were provided two times per week. Dead flies were recorded daily. The median lifespan, the age of 90% mortality (maximum lifespan), and the mortality rate doubling time (MRDT) were calculated.

### Stress resistance dynamic analysis

Male and female flies were collected under CO_2_ anesthesia and aged 1 (young), 4 (mature) and 6 (old) weeks in *Drosophila* narrow vials of 30 flies. Animals were assessed for resistance to hyperthermia (35 °C), oxidative stress (20 mM paraquat, Sigma-Aldrich, USA), and endoplasmic reticulum stress (12 µM tunicamycin, Sigma-Aldrich, USA). Stressful conditions were applied until the end of life. To assess the stress resistance, each fly was maintained in 5 mm glass tubes placed in *Drosophila* Activity Monitors (Trikinetics, USA). Tubes contained a medium composed of 1.3% agarose, 1% sucrose, and either 20 mM paraquat or 12 μM tunicamycin, or no toxic compound (hyperthermia and control). The compounds were introduced to the medium at 45 °C to prevent heat inactivation. The locomotor activity data from individual flies were collected in 30-minute bins and analyzed. Dead flies were identified as flies without movements. The time of the death was accepted at the next 30-minute period after last movement. The survival time for 25%, 50%, 75% and 90% of populations were estimated and survival curves were plotted. The 32 male and female flies were analyzed per each experimental variant in 3 replicates. A total of 96 males and 96 females were analyzed.

### Analysis of fecundity

Fecundity was estimated by the number of eggs laid per day, per female. The pairs of one analyzed female and one *Canton-S* (#64349, Bloomington *Drosophila* Stock Center, USA) wild-type male were placed into 15 ml penicillin vials with agar-yeast nutrient medium colored with activated charcoal. After 24 hours, the flies were removed and the number of eggs was counted. Fecundity was estimated once a week until the age of 10 weeks. Once a month, the *Canton-S* males were replaced by young ones. Between the measurements, flies were housed on a standard food medium in *Drosophila* narrow vials of 15 couples. Experiments were performed in two replicates (a total of 100 females per experimental variant).

### Library preparation and transcriptome sequencing

For transcriptomic analysis, 50 males and females were prepared separately for each experimental variant (control and experimental flies at the age of 1 (young), 4 (mature), and 6 (old) weeks) in 3 replicates. For convenience, we have abbreviated the title of the samples (for example, YFE01 means Young, Female, Experimental flies, 1^st^ replicate). Flies were collected, immediately snap frozen in liquid nitrogen and stored at −80 °C. Total RNA was extracted from 30 flies (10 flies per replicate) using QIAzol Lysis Reagent (Qiagen, Netherlands) with the isopropanol precipitation. All samples were treated with DNase I (Promega, USA). RNA concentration and quality were evaluated using a Qubit 2.0 fluorometer (Life Technologies, USA) and Agilent 2100 Bioanalyzer (Agilent Technologies, USA). Library preparation was carried out by a previously used protocol^59^. Double stranded cDNA library was prepared by using NEBNext® Ultra™ Directional RNA Library Prep kit for Illumina following manufacturer’s protocol from 1 mg of total RNA. The quantity of libraries was determined using the qPCR method by Rotor-Gene 6000 PCR System (Qiagen, USA) according to the manufacturer’s protocol. Primers matched sequences within adapters, flanking an Illumina sequencing library. Before starting qPCR, a control template was selected to measure the libraries for quantification. The quality of libraries was defined using Agilent 2100 Bioanalyzer (Agilent, USA) according to the manufacturer’s protocol. cDNA libraries were normalized to 2 nM, pooled together in equal volumes, and sequenced with 50 bp single-end reads on the HiSeq™2000 platform (Illumina, USA). Illumina HiSeq Analysis Software was used to obtain raw sequencing reads. The sequencing data were stored in FASTQ format. At least 25 million reads were obtained for each pool of flies.

### RNA sequencing data analysis

RNA sequencing data analysis. We used PPLine pipeline to perform quality control, read mapping, and counting^60^. In details, reads were analyzed using FastQC and trimmed with trimmomatic^61^. In order to evaluate efficacy of isolation of polyA fraction and absence of bacterial contamination, 100,000 randomly selected reads from each sample were mapped to *D*. *melanogaster* rRNA genes and bacterial genomes (all strains that had been submitted to NCBI Genome database up to 2015), respectively. Typically, rRNA ratio was 0.3–1.7%. Bacterial contamination did not exceed 0.3%.

The trimmed reads were mapped to the *D*. *melanogaster* genome (assembly BDGP6, Ensembl release 90) using splice-aware STAR aligner^62^. About 90–95% reads were uniquely mapped. Since the samples had demonstrated different RNA integrity numbers (RIN, 5…8) in order to compare gene expression levels, we took into account 3′-tail bias as described in the original research of Sigurgeirsson *et al*.^63^ with some modifications. Without 3′-bias adjustment, one should expect false-positive overexpression of short transcripts (for example, genes of mitochondrial proteins or ribosomal proteins) and under-expression of genes with long transcripts and encoded proteins (for example, multi-domain transmembrane proteins interacting with extracellular matrix).

Firstly, we analyzed read coverage across transcript length using a modified version of geneBody_coverage.py script, a part of RSeQC package^64^. In the samples with lower RIN numbers and more degraded RNA, we observed rapid falling down of read coverage level after 750–1000 bp from 3′-tail. The coverage in the region of 0–750 bp from 3′-tail was almost identical across the samples.

Secondly, we quantified transcripts using RSEM^65^ and identified the most abundantly expressed alternative transcript for each gene. The other transcripts were discarded. Then, the transcripts were truncated to the length of 750 bp from 3′-end and new GTF file was generated. Finally, using the derived GTF gene model, we quantified reads using featureCounts from Subread package^66^. This procedure allowed us to significantly reduce the dependence of the observed expression level fold changes (FC) on the transcript lengths between the samples with higher and lower RINs.

In order to even more effectively eliminate the factor of 3′-bias, we proceed the following way. All the genes were split into 10 bins depending on the average gene transcript length. Then, within each bin, we normalized read counts using TMM (trimmed mean of M-values) method from edgeR^67^. A similar procedure was also performed to eliminate the dependency on the ‘absolute’ gene expression level (in terms of read counts per million, CPM). This bias may also occur when comparing samples with different RINs and different RNA concentration. Finally, the adjusted read pseudo-counts were analyzed by edgeR using exact test and quasi-likelihood ratio F-test^67^.Before the analysis, we filtered genes by expression level (CPM > 1.0 for at least 50% samples of the smallest group).

Gene Ontology, KEGG, Reactome enrichment tests for top-50, 100, 200, 500 and 1000 differentially expressed genes (DEG; p < 0.05) were performed using topGO and clusterProfiler Bioconductor packages^68^. The results were merged between the top DEG lists. Additionally, we performed enrichment test for DEG lists which were derived without CPM filtration, relying only on edgeR’s F-test p-value threshold (0.05). KEGG pathways visualization was performed using the modification of pathview package^69^ as described earlier^70^.

### Statistical analysis

To compare the statistical differences in survival functions and median lifespan between control and experimental groups, the modified Kolmogorov-Smirnov and log-rank test were used, respectively^71,72^. A Wang-Allison test was used to estimate the differences in the age of 90% mortality^73^. To assess the statistical significance of differences in resistance to stress factors, the Fisher’s exact test was used^74^. Statistical analyses of the data were performed using STATISTICA software, version 6.1 (StatSoft, USA), R, version 2.15.1 (The R Foundation) and OASIS 2 (Online Application for Survival Analysis 2)^75^.

## Supplementary information

Supplementary figures

Supplementary Table 1

Supplementary Table 4

## Data Availability

The authors declare that the data supporting the findings reported in this manuscript are available within the article and the Supplementary Information fIles or from the corresponding author upon request. The sequencing data are available through the NCBI Sequence Read Archive (Project ID PRJNA474000).
