# Supplementary material for: Transcriptome Analysis of Long-lived Drosophila melanogaster E(z) Mutants Sheds Light on the Molecular Mechanisms of Longevity
Source: Sci Rep. 2019 Jun 24;9:9151. doi: 10.1038/s41598-019-45714-x (PMC6591219; doi:10.1038/s41598-019-45714-x)
Supplement: Supplementary file 1 — Supplementary figures [file 41598_2019_45714_MOESM1_ESM.docx]

**Transcriptome Analysis of Long-lived *Drosophila melanogaster E(z)* Mutants Sheds Light on the Molecular Mechanisms of Longevity**

Alexey A. Moskalev^1,2,*^, Mikhail V. Shaposhnikov^2^, Nadezhda V. Zemskaya^2^, Liubov А. Koval^2^, Eugenia V. Schegoleva^2^, Zulfiya G. Guvatova^1^, George S. Krasnov^1^, Ilya A. Solovev^2^, Maksim A. Sheptyakov^3^, Alex Zhavoronkov^4^, Anna V. Kudryavtseva^1^

^1^ Engelhardt Institute of Molecular Biology, Russian Academy of Sciences, Moscow, Russia

^2^ Institute of Biology of Komi Science Center of Ural Branch of RAS, Syktyvkar, Russia

^3^ Moscow Institute of Physics and Technology, Dolgoprudny, Russia

^4^ Insilico Medicine, Baltimore, USA

* Correspondence: amoskalev@list.ru

**Supplementary information**

**Supplementary figures**


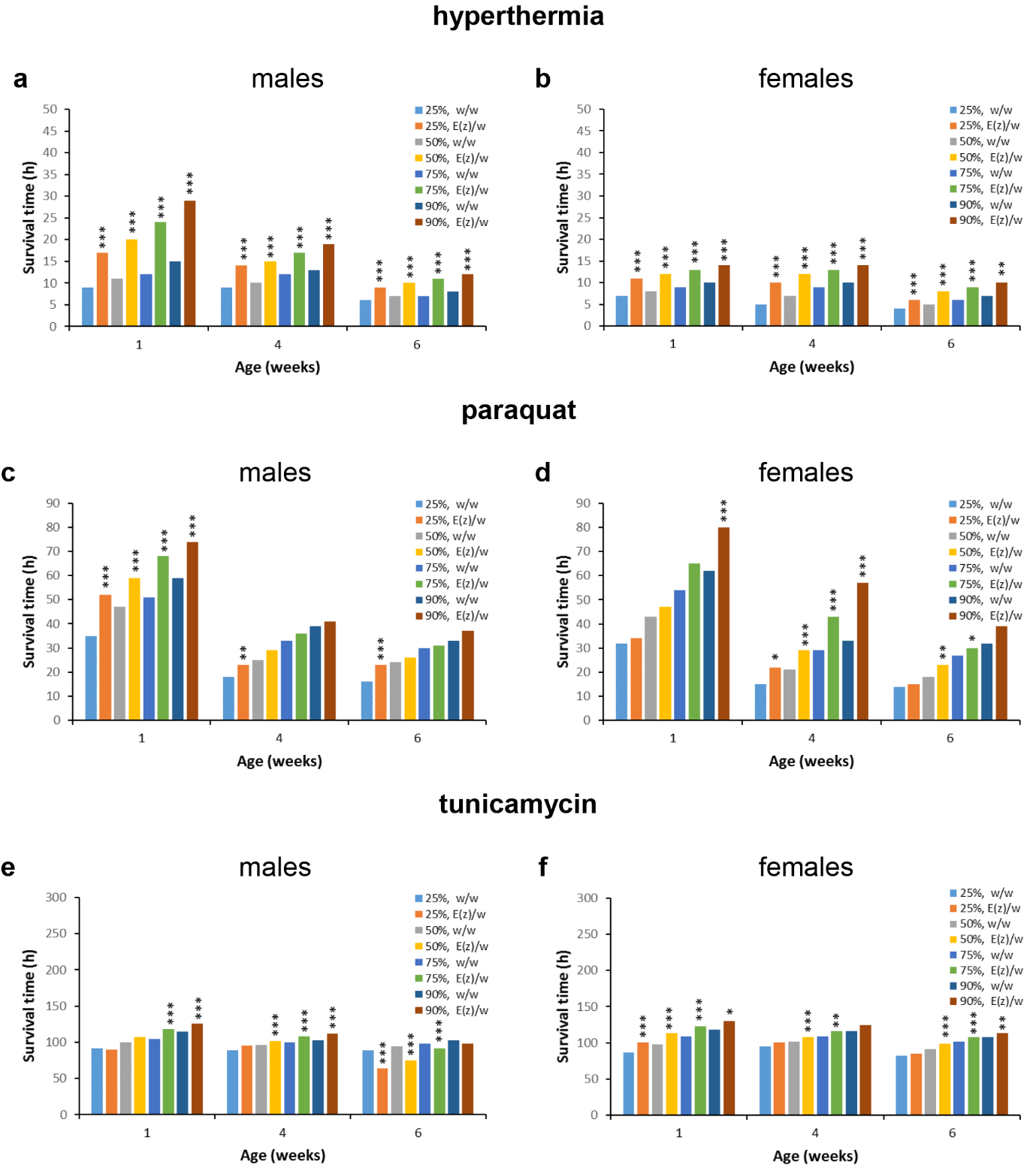


**Supplementary Figure 1.** Influence of heterozygous mutation in *E(z)* on (**a,c,e**) male and (**b,d,e**) female resistance to (**a,b**) hyperthermia, (**c,d**) paraquat and (**e,f**) tunicamycin at the ages of 1, 4 and 6 weeks. The survival time for 25%, 50%, 75% and 90% of populations are estimated. *p<0.05, **p<0.01, ***p<0.001, Fisher’s exact test.

**
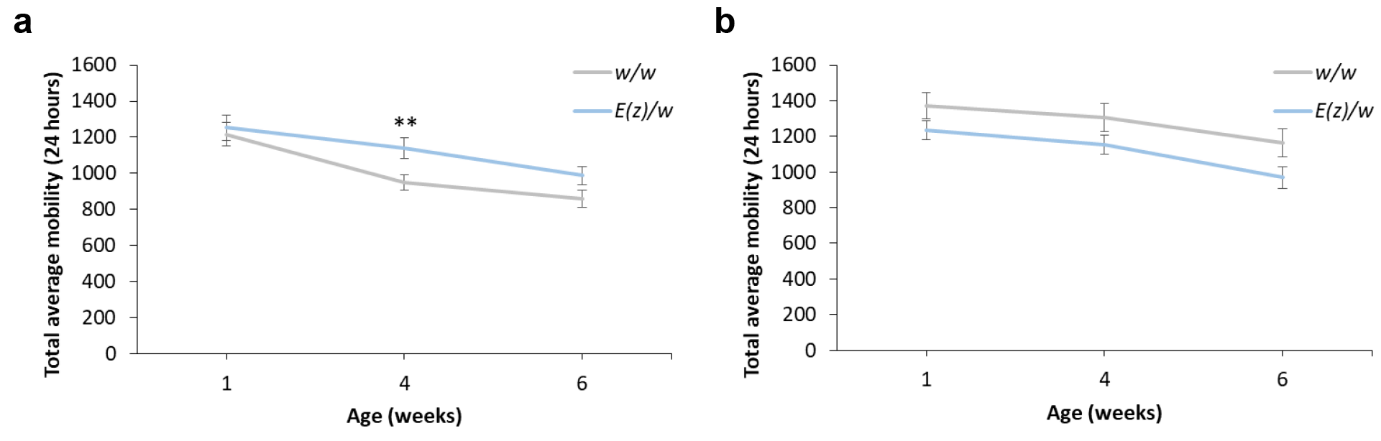
**

**Supplementary Figure 2.** Influence of heterozygous mutation in *E(z)* on age-dependent dynamics of total daily locomotor activity of males (**a**) and females (**b**). The error bars show standard errors. **p<0.01, t-Student test.

**Supplementary figures**


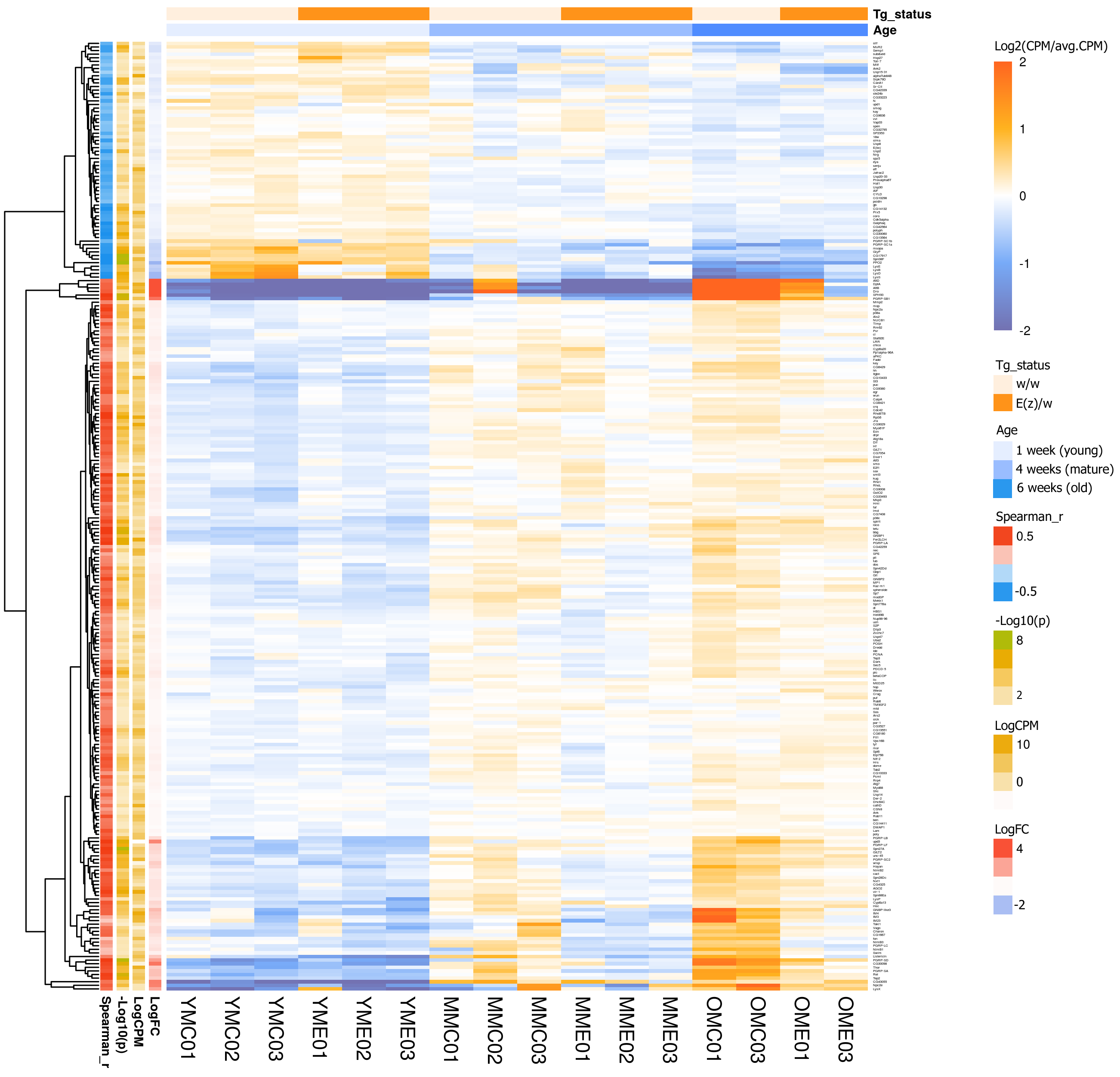


**Supplementary Figure 3.** Heatmap illustrating age-dependent changes in expression level of genes involved in the immune response, including the defense response, the innate immune response, the inflammatory response etc. (according to GO database). DE genes in male’s groups are presented(p<0.05).


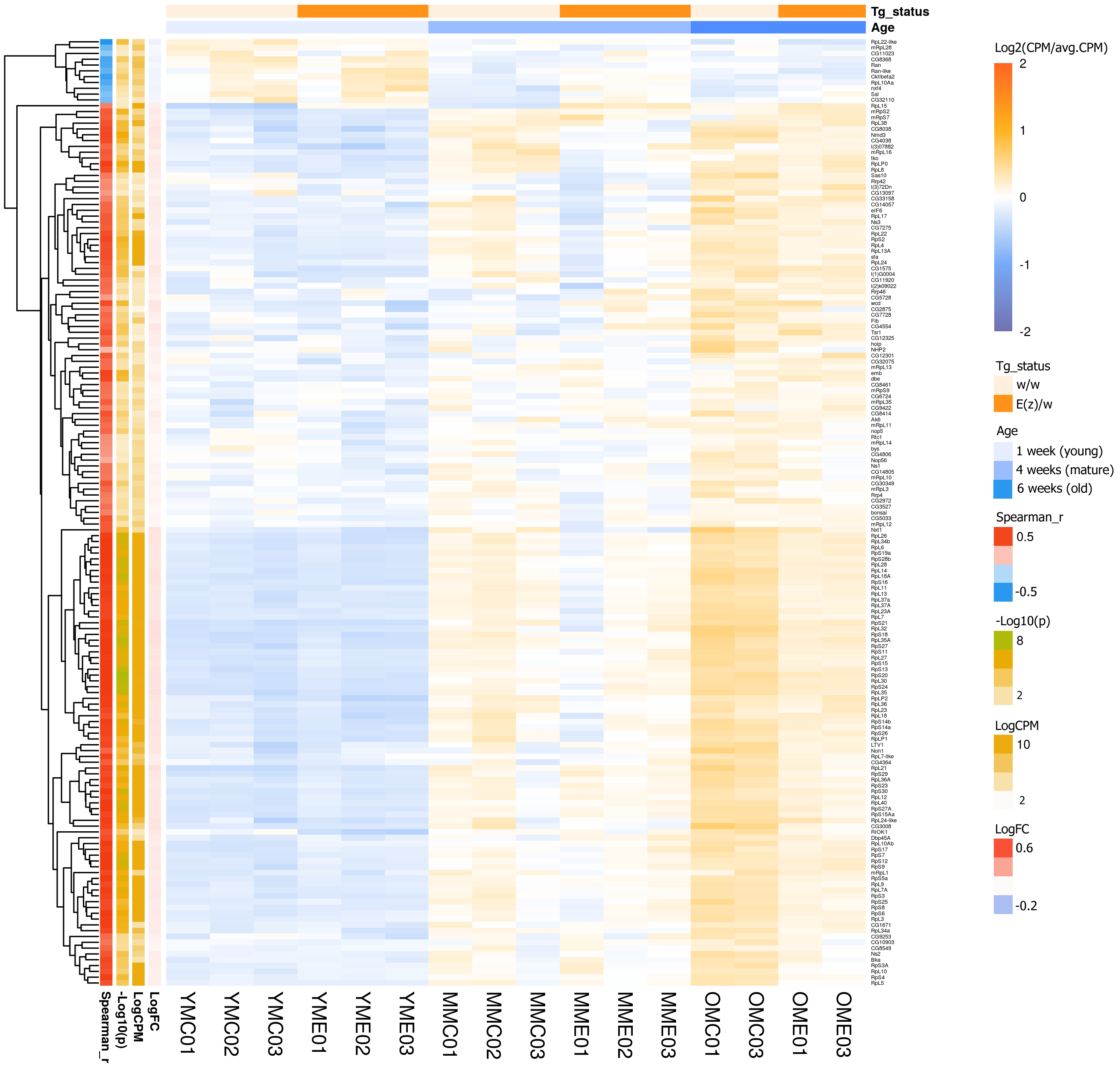


**Supplementary Figure 4.** Heatmap illustrating age-dependent changes in expression level of genes associated with ribosome biogenesis (according to GO and KEGG pathway database). DE genes in male’s groups are presented(p<0.05).

**
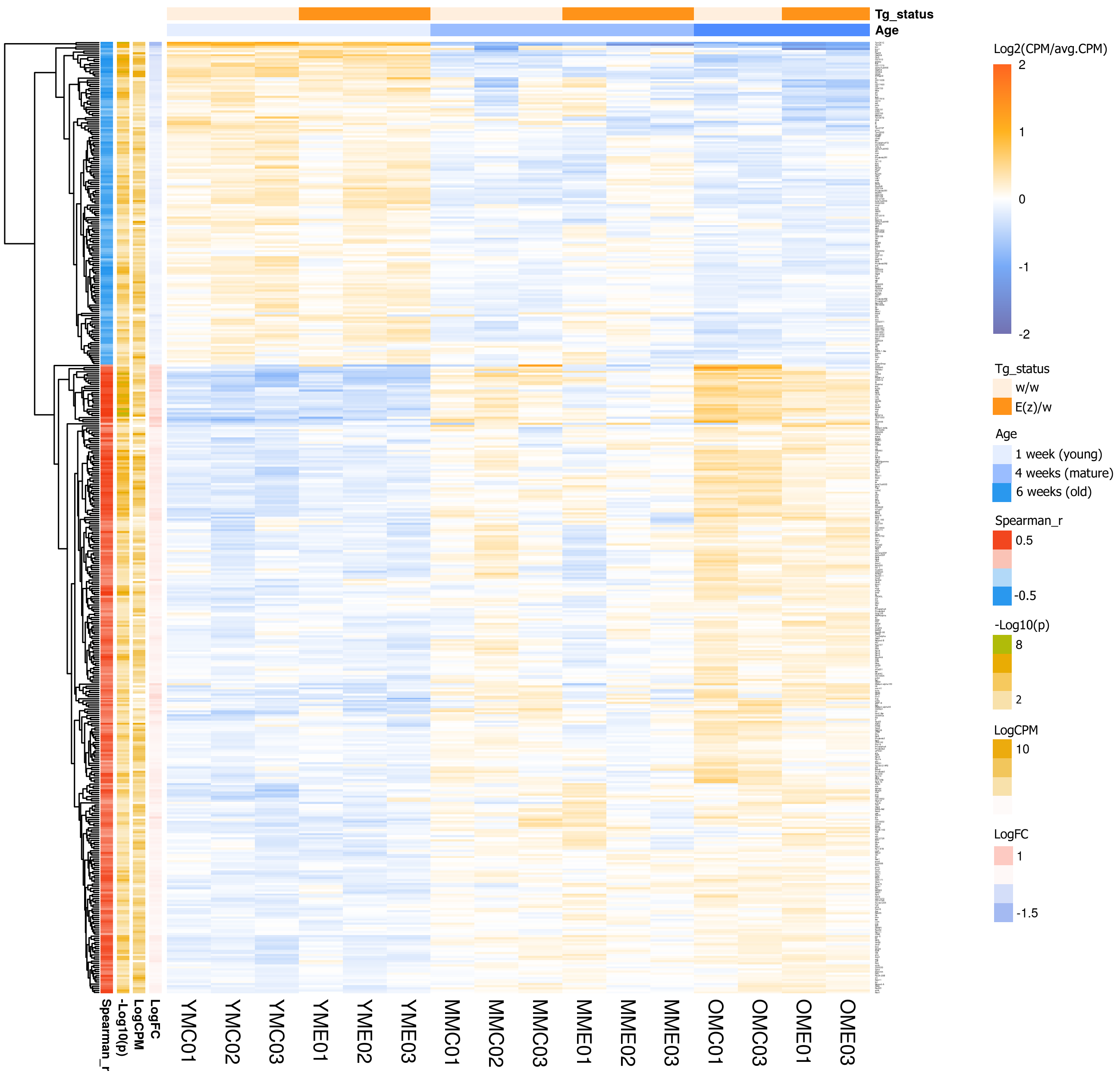
**

**Supplementary Figure 5.** Heatmap illustrating age-dependent changes in expression level of genes involved in the cell cycle (according to GO and KEGG pathway database). DE genes in male’s groups are presented(p<0.05).

**
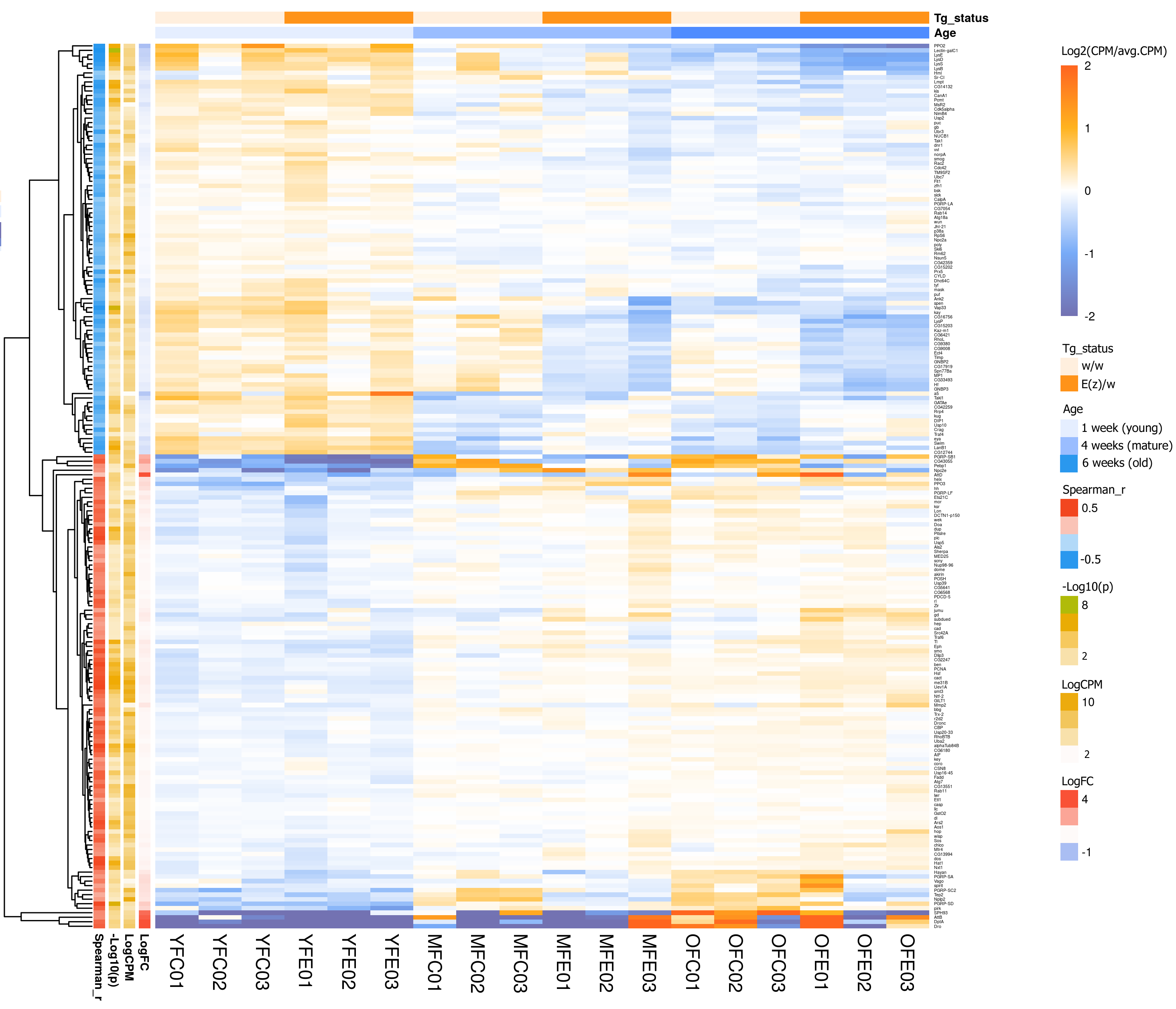
**

**Supplementary Figure 6.** Heatmap illustrating age-dependent changes in expression level of genes involved in the immune response, including the defense response, the innate immune response, the inflammatory response etc. (according to GO database). DE genes in female’s groups are presented(p<0.05).


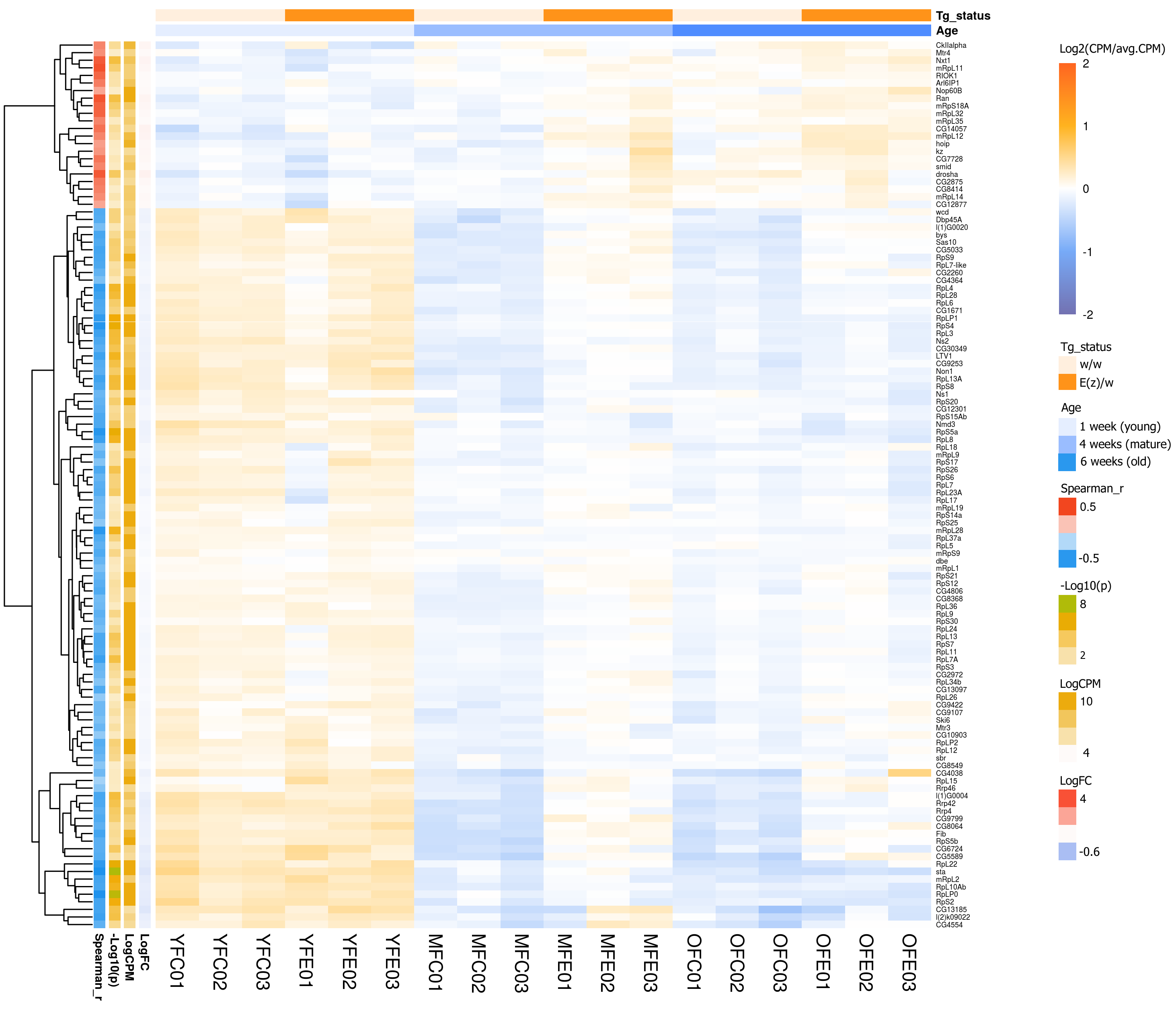


**Supplementary Figure 7.** Heatmap illustrating age-dependent changes in expression level of genes associated with ribosome biogenesis (according to GO and KEGG pathway database). DE genes in female’s groups are presented(p<0.05).

**
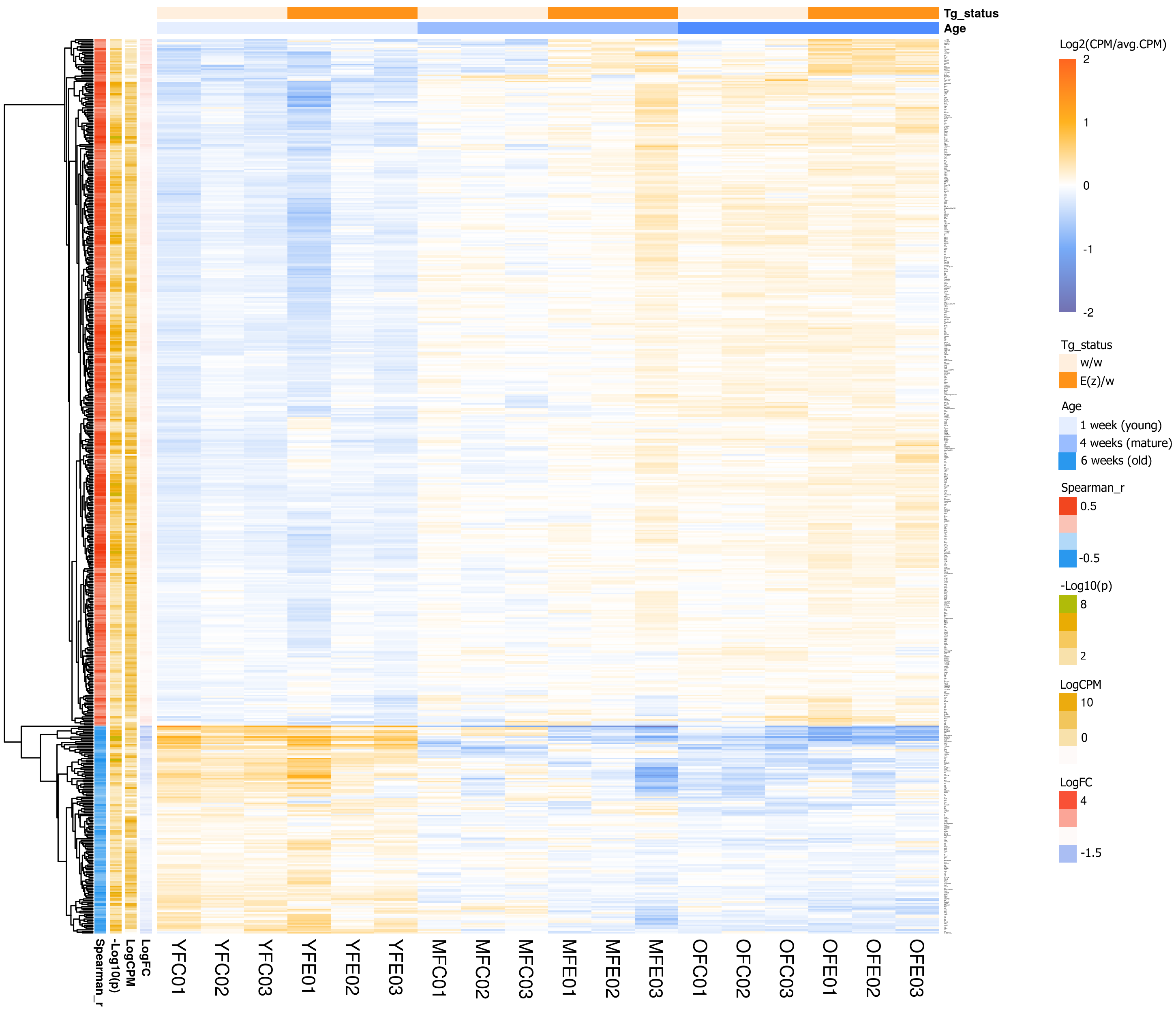
**

**Supplementary Figure 8.** Heatmap illustrating age-dependent changes in expression level of genes involved in the cell cycle (according to GO and KEGG pathway database). DE genes in male’s groups are presented(p<0.05).

**
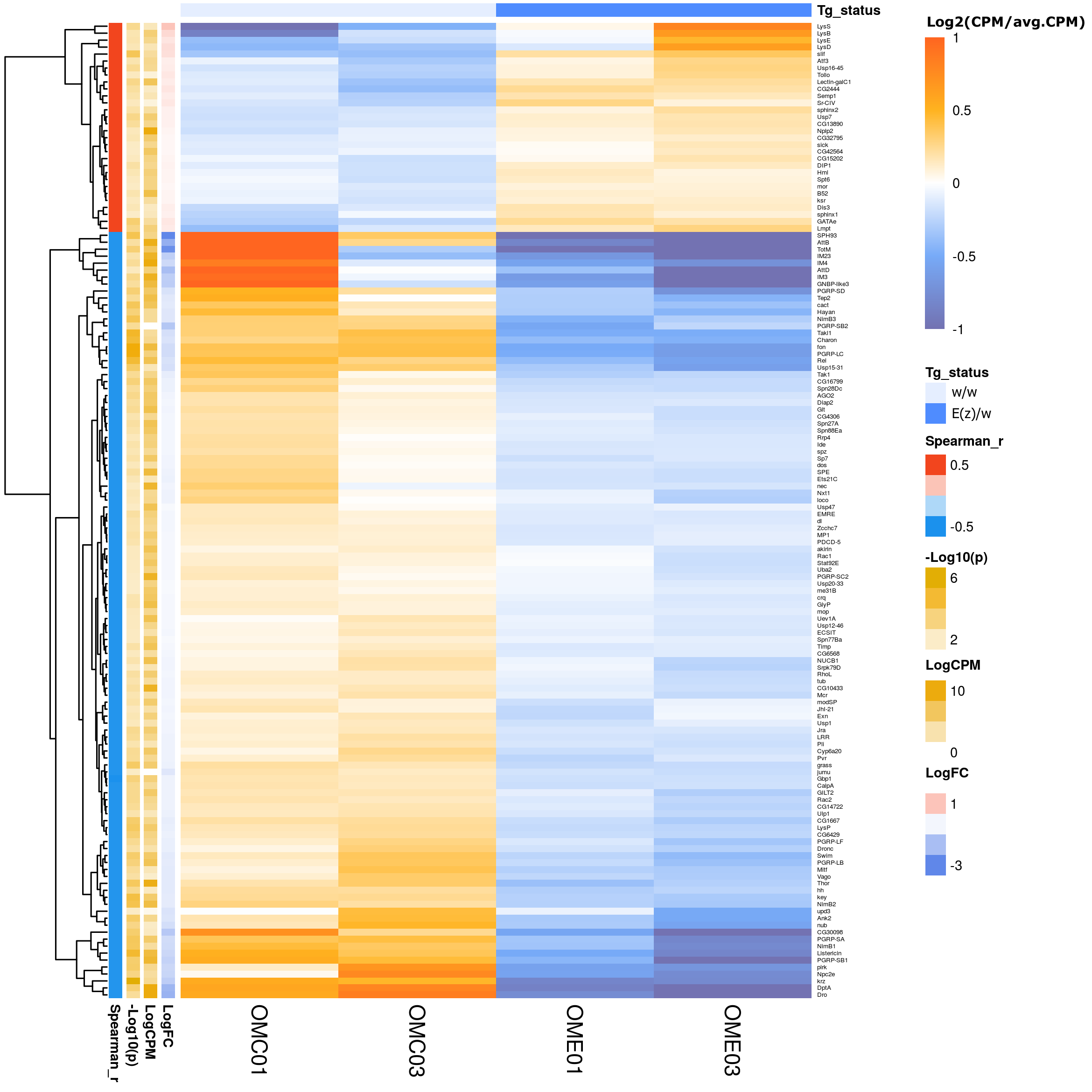
**

**Supplementary Figure 9.** Heatmap illustrating the gene expression differences in "Old" *E(z)/w* mutant males versus *w/w* control "Old" ones. Genes with ontologies related to immune response are presented, including the defense response, the innate immune response, the inflammatory response etc. (according to GO database) (p<0.05).

**
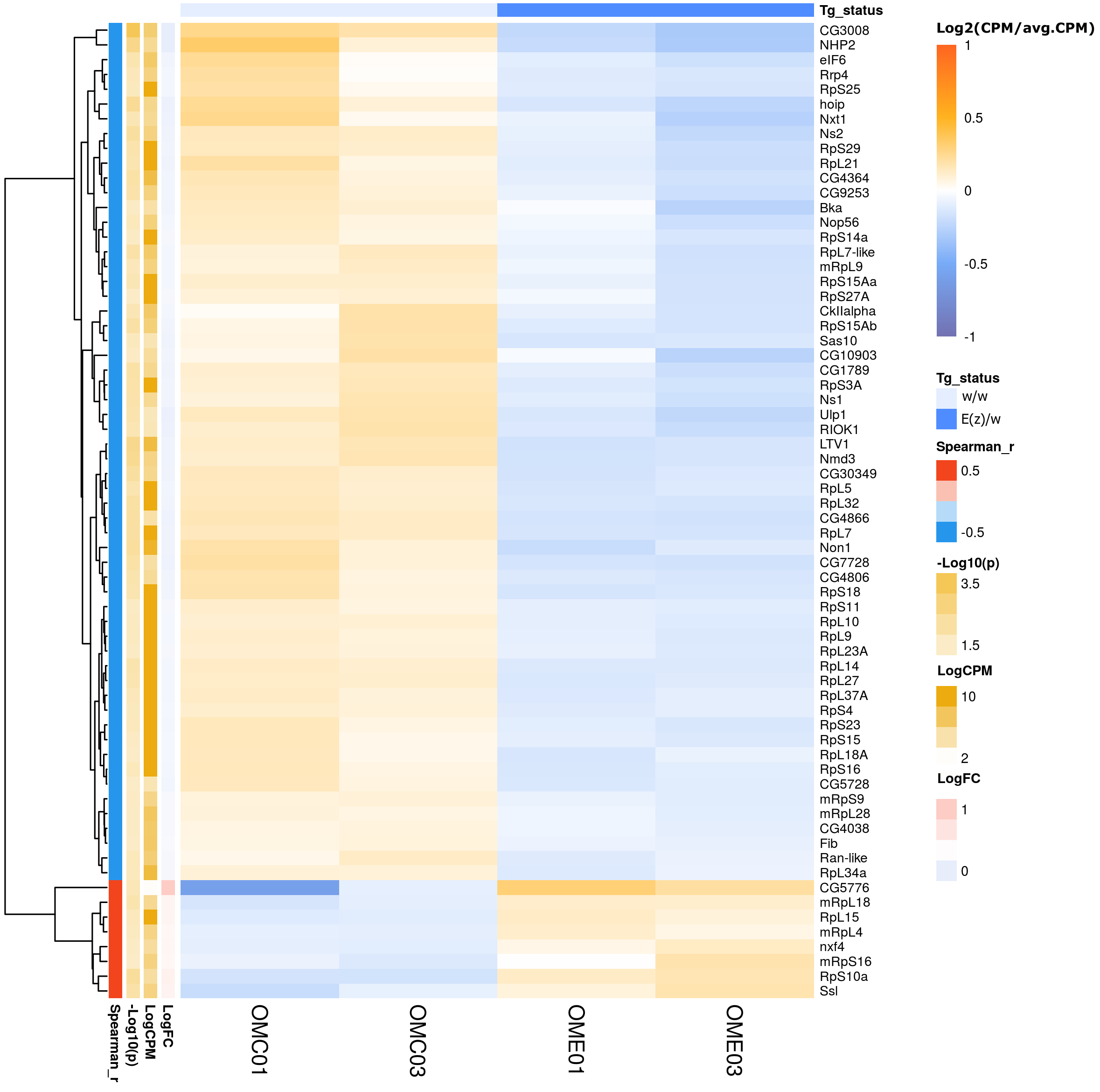
**

**Supplementary Figure 10.** Hitmap illustrating the gene expression differences in "Old" *E(z)/w* mutant males versus *w/w* control "Old" ones. Genes with ontologies related to ribosome biogenesis are presented (according to GO and KEGG pathway database) (p<0.05).


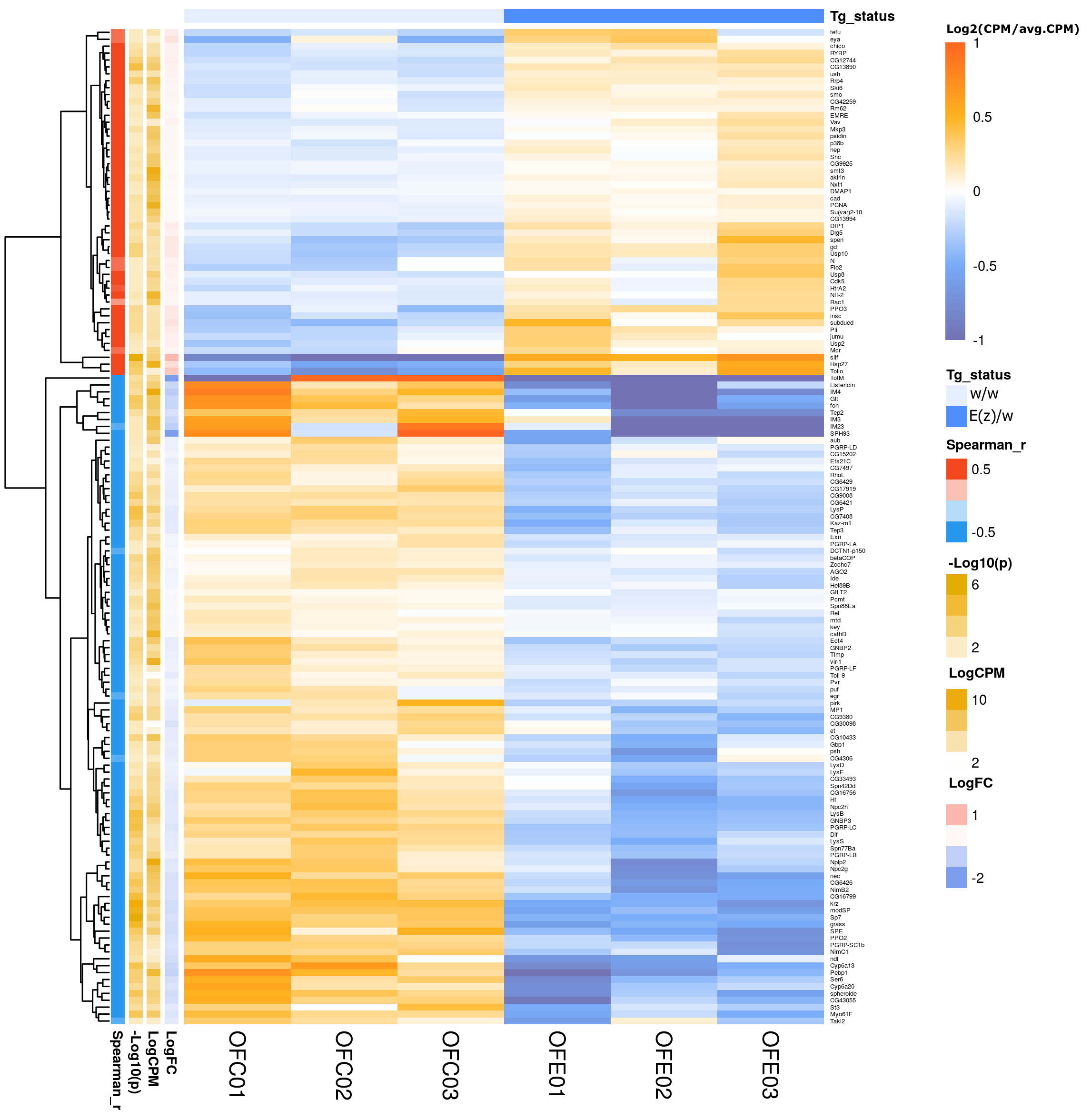


**Supplementary Figure 11.** Heatmap illustrating the gene expression differences in "Old" *E(z)/w* mutant females versus *w/w* control "Old" ones. Genes with ontologies related to immune response are presented, including the defense response, the innate immune response, the inflammatory response etc. (according to GO database) (p<0.05).

**
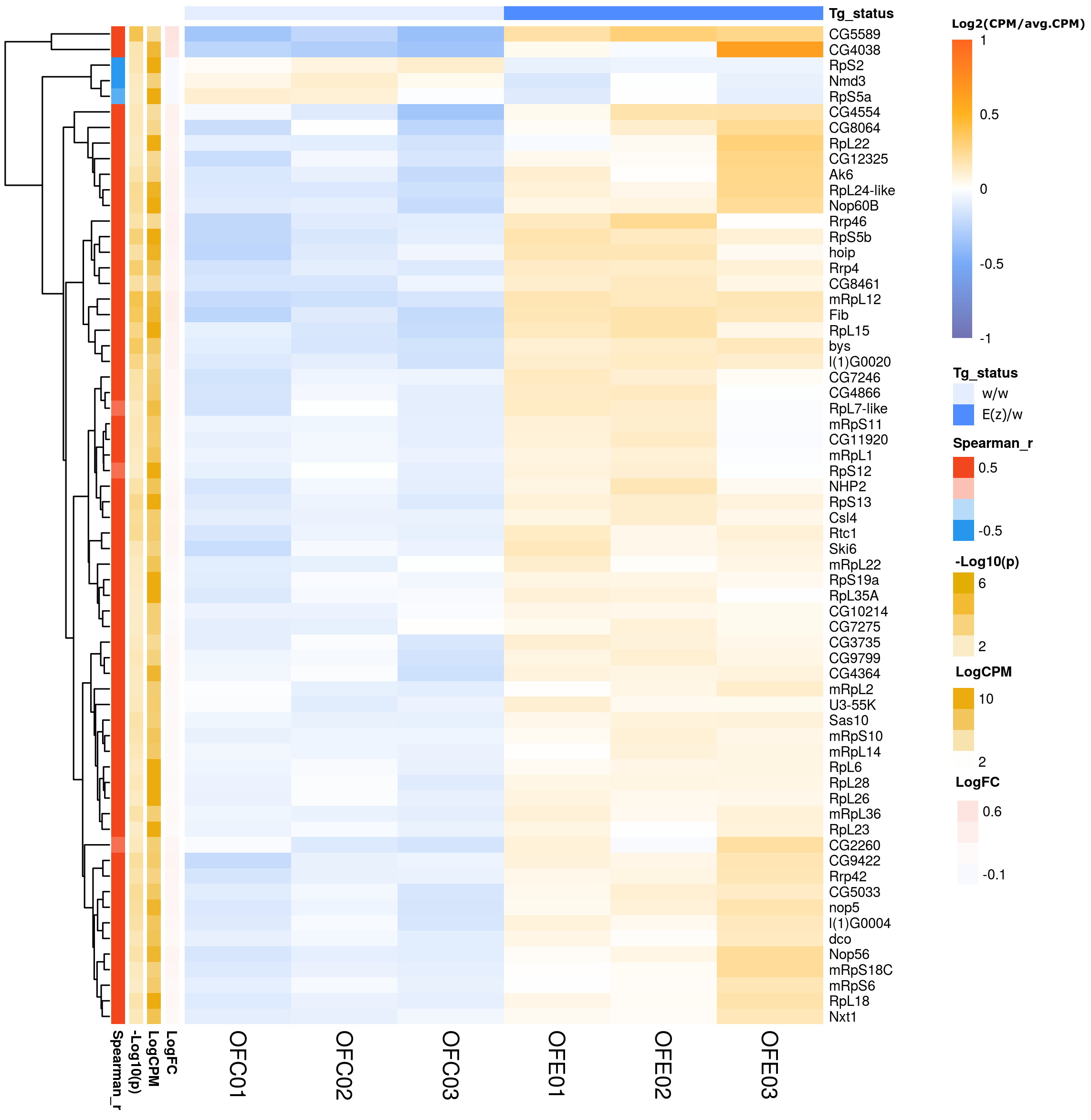
**

**Supplementary Figure 12.** Heatmap illustrating the gene expression differences in "Old" *E(z)/w* mutant females versus *w/w* control "Old" ones. Genes with ontologies related to ribosome biogenesis are presented (according to GO and KEGG pathway database) (p<0.05).

**Supplementary tables**

| Gene ID | Symbol | Biotype | Name | LogFC |  |
| --- | --- | --- | --- | --- | --- |
| FBgn0014076 | *Vm32E* | protein_coding | Vitelline membrane 32E | -1.17 |  |
| FBgn0086266 | *Vm26Ac* | protein_coding | Vitelline membrane 26Aac | -0.93 |  |
| FBgn0085362 | *Vml* | protein_coding | Vitelline membrane-like | -0.91 |  |
| FBgn0003979 | *Vm26Aa* | protein_coding | Vitelline membrane 26Aa | -0.77 |  |
| FBgn0003980 | *Vm26Ab* | protein_coding | Vitelline membrane 26Ab | -0.64 |  |

**Supplementary Table 2.** Differently expressed genes of Vitelline membrane associated with a mutation in a *E(z)* gene. For *Vm32E* and *Vm26Ac* p-values was less than 0.05,quasi-likelihood F-test (QLF).

|  |  |  | Males | Females |
| --- | --- | --- | --- | --- |
| Gene ID | Symbol | Name | (LogFC) | (LogFC) |
| FBgn0041581 | *AttB* | Attacin-B | -2.36 | 1.27 |
| FBgn0012042 | *AttA* | Attacin-A | -2.57 | 1.6 |
| FBgn0041579 | *AttC* | Attacin-C | -2.09 | -0.02 |
| FBgn0034407 | *DptB* | Diptericin B | -1.91 | 0.7 |
| FBgn0004240 | *DptA* | Diptericin A | -2.29 | 1.75 |
| FBgn0000277 | *CecA2* | Cecropin A2 | -1.86 | 1.48 |
| FBgn0000276 | *CecA1* | Cecropin A1 | -1.02 | 1.99 |
| FBgn0000279 | *CecC* | Cecropin C | -2.52 | 1.53 |

**Supplementary Table 3.** Down- and up-regulated genes of antimicrobial peptides (AMPs) associated with a mutation in a *E(z)* gene for males and females groups. p<0.05, quasi-likelihood F-test (QLF).

| Gene ID | Symbol | Biotype | LogFC | p (QLF test) | FDR (QLF test) | p (LR test) | p (ex. test) | p (wilcoxon) | p (t-test) |
| --- | --- | --- | --- | --- | --- | --- | --- | --- | --- |
| FBgn0260463 | *Unc-115b* | protein_coding | 7.28 | 9.26E-16 | 1.19E-11 | 3.30E-152 | 2.60E-151 | 3.29E-07 | 1.12E-12 |
| FBgn0053105 | *p24-2* | protein_coding | 6.8 | 5.20E-15 | 3.35E-11 | 3.01E-82 | 2.01E-81 | 2.70E-07 | 1.26E-09 |
| FBgn0261575 | *tobi* | protein_coding | 8.71 | 4.19E-14 | 1.80E-10 | 5.22E-39 | 1.19E-38 | 7.01E-07 | 0.0001 |
| FBgn0260874 | *Ir76a* | protein_coding | 3.15 | 3.78E-13 | 1.22E-09 | 1.85E-28 | 2.48E-28 | 7.03E-07 | 1.65E-06 |
| FBgn0035941 | *CG13313* | protein_coding | 5.07 | 6.89E-13 | 1.77E-09 | 2.22E-44 | 4.32E-44 | 6.73E-07 | 2.56E-12 |
| FBgn0037975 | *CG3397* | protein_coding | 6.97 | 2.85E-08 | 2.16E-05 | 3.06E-10 | 6.41E-10 | 2.14E-07 | 8.07E-07 |
| FBgn0036648 | *CG4098* | protein_coding | 3.23 | 6.32E-08 | 3.54E-05 | 1.95E-12 | 2.59E-12 | 1.41E-05 | 0.0005 |
| FBgn0054031 | *CG34031* | protein_coding | 4.61 | 4.44E-08 | 2.88E-05 | 9.28E-14 | 1.44E-13 | 0.0002 | 0.0006 |
| FBgn0037974 | *CG12224* | protein_coding | 3.28 | 8.83E-07 | 0.0003 | 2.51E-10 | 3.22E-10 | 3.82E-06 | 0.002 |
| FBgn0036471 | *CG13460* | protein_coding | 3.84 | 7.29E-06 | 0.002 | 1.58E-08 | 2.33E-08 | 2.20E-06 | 0.001 |
| FBgn0052379 | *CG32379* | protein_coding | -2.02 | 1.12E-09 | 1.32E-06 | 2.61E-15 | 3.02E-15 | 2.36E-06 | 1.47E-05 |
| FBgn0264987 | *CR44138* | ncRNA | -2.18 | 3.82E-06 | 0.001 | 2.23E-08 | 2.57E-08 | 0.0001 | 0.001 |
| FBgn0262104 | *CG42857* | protein_coding | -2.91 | 0.0002 | 0.03 | 5.84E-06 | 7.18E-06 | 0.001 | 0.004 |

**Supplementary Table 5.** Down- and up-regulated genes associated with a mutation in a *E(z*) gene. Significant DE genes with |LogFC|>2 are represented. p<0.05, FDR<0.05 quasi-likelihood F-test (QLF).

| Gene ID | Symbol | Young  (LogFC) | Mature  (LogFC) | Old  (LogFC) | Males young  (LogFC) | Males mature  (LogFC) | Males old  (LogFC) | Females young  (LogFC) | Females mature  (LogFC) | Females old  (LogFC) |
| --- | --- | --- | --- | --- | --- | --- | --- | --- | --- | --- |
| FBgn0031701 | *TotM* | 1.75 | -5.19 | -3.05 | 1.85 | -5.77 | -3.17 | 1.18 | -3.7 | -2.84 |
| FBgn0028396 | *TotA* | 0.81 | -2.79 | -2.35 | 0.71 | -2.6 | -2.15 | 0.96 | -3.42 | -2.84 |
| FBgn0044812 | *TotC* | 1.57 | -4.76 | -3.29 | 1.49 | -4.71 | -3.3 | 1.36 | -5.08 | -3.43 |
| FBgn0044810 | *TotX* | 0.44 | -1.85 | -1.26 | 0.47 | -1.68 | -0.9 | -0.02 | -2.43 | -1.77 |

**Supplementary Table 6.** Differently expressed genes of the Turandot family compared with the corresponding control groups. p<0.05, quasi-likelihood F-test (QLF).

| Gene ID | Symbol | Biotype | Name | LogFC |
| --- | --- | --- | --- | --- |
| FBgn0039593 | *Sid* | protein_coding | Stress induced DNase | -1,46 |

**Supplementary Table 7.** The expression level of Sid gene decrease in *E(z)* mutants. p<0.05, quasi-likelihood F-test (QLF).

**Supplementary methods**

**Locomotor activity analysis.** The age-dependent dynamics of spontaneous locomotor activity was analyzed by the *Drosophila* Locomotor Activity Monitor (Trikinetics, USA). Locomotor activity of 1, 4, and 6-week-old individual flies was recorded. The data were collected during 24 hours and represented as average total daily locomotor activity. The 16 male and female flies were analyzed per each experimental variant in three replicates. A total of 48 males and 48 females were analyzed.
